# Utility of Two-Dimensional Difference Gel Electrophoresis in Diagnosis of Multiple Sclerosis

**DOI:** 10.3390/diagnostics8030044

**Published:** 2018-07-05

**Authors:** Michael Auer, Harald Hegen, Dagmar Rudzki, Georg Golderer, Florian Deisenhammer

**Affiliations:** 1 Department of Neurology, Medical University of Innsbruck, Innrain 66, 6020 Innsbruck, Austria; harald.hegen@i-med.ac.at (H.H.); dagmar.rudzki@tirol-kliniken.at (D.R.); florian.deisenhammer@tirol-kliniken.at (F.D.); 2 Division of Biological Chemistry, Medical University of Innsbruck, Innrain 80-83, 6020 Innsbruck, Austria; georg.golderer@i-med.ac.at

**Keywords:** 2D-DIGE, diagnosis, electrophoresis, multiple sclerosis, protein

## Abstract

Two-dimensional difference gel electrophoresis (2D-DIGE) has been used for identification of possible biomarkers in the cerebrospinal fluid (CSF) of multiple sclerosis (MS) patients. However, in different studies inconsistent results have been obtained. We wanted to analyze the diagnostic value of 2D-DIGE in early MS patients by comparing protein patterns between single and pooled samples of MS patients and controls. CSF samples of 20 MS patients and 10 control subjects were processed with 2D-DIGE. The so obtained protein patterns were analyzed with DeCyder 6.5 software, whereby we described variation of patterns presented in one gel as well as between different gels. Even when running single samples of patients of the same group in one gel, variation of protein patterns was high. The number of identified spots with different protein level varied between 4 and 30, depending on which sample batches were compared. We did not find a consistent pattern throughout all possible batch combinations. The inter-individual variation of protein expression as well as the susceptibility of 2D-DIGE for methodological variations makes use of 2D-DIGE as a diagnostic tool for MS and for detection of possible candidate biomarkers difficult, since detected proteins vary depending on which samples are compared.

## 1. Introduction

Multiple sclerosis (MS) is a chronic inflammatory disease of the central nervous system with vast heterogeneity between individual patients and different immunologic processes involved [1]. The exact immunologic mechanisms leading to demyelination are still poorly understood and various approaches to find candidate genes, proteins or cells were of little success so far. One of many methods in biomarker research is two-dimensional gel electrophoresis (2DE) [2]. This high-resolution method allows separation of single proteins by their molecular weight and charge [3,4]. After separation proteins can be picked from 2DE gels and identified e.g., by mass spectrometry. In MS, proteins of the cerebrospinal fluid (CSF) may be of major interest in order to identify possible candidate proteins which are up- or downregulated in comparison to healthy subjects [5]. There are some studies investigating CSF-proteins of MS patients by 2DE or two-dimensional difference gel electrophoresis (2D-DIGE) [5,6,7,8,9,10,11,12,13]. 2D-DIGE provides the advantage of performing analyses on a reduced number of gels with comparison of samples in the same gel run under the same conditions and with a consistent pool-standard throughout all gels [14,15,16]. However, results of these approaches were inconsistent so far, and the results of previous experiments could not be confirmed by later repetition of different research groups. Additionally, most authors used pooled CSF in order to obtain sufficient protein concentrations by merging CSF-samples of more patients in one gel. It is not clear if pooled samples give the same results as averaging single runs. The present study aimed to compare CSF-samples of single patients versus pooled samples as well as MS-CSF versus control-CSF of single patients and pooled samples by 2D-DIGE. In contrast to previous studies, we did not aim to identify single proteins by spot-picking, but we focused on protein patterns performing analyses with a statistical software in order to describe the accordance of these patterns throughout all samples and to analyze the consistency in obtaining specific protein spots.

## 2. Materials and Methods

### 2.1. Sample Collection

CSF was obtained by lumbar puncture for routine examination and continuously stored at −80 °C immediately after centrifugation for 10 min at 1500× *g* at room temperature. All patients signed an informed consent form for anonymous use of residual samples which was approved by the ethics committee. We chose CSF of twenty patients who underwent lumbar puncture at the first clinical manifestation of relapsing remitting multiple sclerosis, i.e., clinically isolated syndrome (CIS), whereby diagnose was confirmed by fulfilling McDonald 2010 [17] MRI criteria for definite MS at time of sampling or in the further disease course. For controls, we chose ten patients fulfilling the criteria of symptomatic controls [18].

### 2.2. Assay

First, CSF samples were concentrated and desalted. For this, precooled (4 °C) Ultrafree MC 500 centrifugal filter units (UFC3BCC00, Millipore, Billerica, MA, USA) were used. The centrifuge was precooled at 4 °C as well. Then, 500 µL of the de-frozen CSF was transferred to the filter units and centrifuged at 14,000× *g* for 10 min. This process was repeated six times, consistently using the same filter unit, in order to obtain 60–70 µL of concentrated and protein-enriched CSF. Additionally, all MS samples and, separately, all control samples were pooled (500 µL per patient, and together concentrated as described above) in order to obtain a pool of MS and controls, which was later used as standard on the DIGE-gels and for separate analyses.

Thereafter, the CSF was cleaned using GE 2D cleanup kit (GE Heathcare, Little Chalfont, UK) with provided buffers according to manufacturer’s manual. The so obtained protein pellet was dissolved in DIGE labeling buffer (5.4 g urea, 400 mg CHAPS, 36.4 mg tris(hydroxymethyl)-aminomethane (tris) diluted in distilled water to a volume of 10 mL, then adjusted to a pH of 8.5 with 1 M HCl).

We did not deplete albumin and immunoglobulins since this led to a marked decrease of concentration of other proteins in the CSF as well in a test-run leading to protein concentrations insufficient to obtain analyzable spots in the 2D-DIGE.

A calculated quantity (protein quantification made by trichloroacetic acid precipitation and photometric measurement using Evolution 201 spectrophotometer from Thermo Scientific, Waltham, MA, USA) of the concentrated and cleaned CSF corresponding to 50 µg protein per sample was diluted in 8.33 µL labeling buffer. Then, 1 µL of the CyDye DIGE Fluors (minimal dyes for 2D-DIGE, Amersham, GE Helthcare, Little Chalfont, UK) were added in order to label samples with Cy2 (pooled samples as standard), Cy3 and Cy5 per gel one each. The samples were incubated on ice under light exclusion for 30 min, followed by addition of 2.4 µL 10 mM lysine as stopping solution, again incubated on ice in the dark for 15 min. After fluors labeling, couples of three samples were merged and rehydrated in 305 µL lysis buffer (4.8 g urea, 400 mg CHAPS, 200 µL Pharmalyte (pH 3–10, Sigma-Aldrich, St. Louis, MO, USA) and 200 mg dithiothreitole (DTT) diluted in distilled water to a volume of 10 mL).

With this solution of DIGE labelled protein in lysis buffer we incubated Immobile DryStrips (18 cm, pH 3–10, Amersham, GE Healthcare, Little Chalfont, UK) over night, covered with 2–3 mL mineral oil in a plastic tray. After rehydration, strips were transferred into the focusing tray and isoelectric focusing (IEF) was conducted with Protean^®^ IEF-cell (BioRad, Hercules, CA, USA) using the CSF-program provided by the manufacturer (90 min at 500 V and rapid voltage slope, 90 min at 1000 V and rapiv slope, 2 h at 8000 V and slow slope, 4 h at 8000 V and rapid slope, 30 min at 500 V and rapid slope). After IEF the strips were washed in 1× SDS buffer (6 g tris, 28.8 g glycine and 1 g sodium dodecyl sulfate (SDS) diluted in distilled water to a volume of 1000 mL).

The SDS polyacrylamide gel electrophoresis (SDS-PAGE) was prepared as follows: For the gels (quantities for 6 gels) we merged 150 mL 40% bis-acrylamide solution and 150 mL tris-HCl solution with pH 8.8 (182 g tris with distilled water to a volume of 1000 mL, titrated to pH 8.8 with 1 M HCl) with 600 mL distilled water. This batch solution was degassed for 30 min, then cooled on ice for additional 15 min. Subsequently, we added 6 mL 10% SDS (10 g SDS with distilled water to 10 mL), 6 mL 10% ammoniumperoxidsulfate (APS) (1 g APS plus 9 mL distilled water) and 1 mL 10% tetramethylethylendiamine (TEMED) (100 µL TEMED plus 900 µL distilled water) in order to obtain the final gel solution. The gels were casted between two glass plates.

Before SDS-page the strips were equilibrated with SDS equilibration buffer (6.7 mL tris-HCl solution with pH 8.8, 73 mg urea, 69 mL 87 vol % glycerine, 4 g SDS, 400 µL 1% bromphenol solution as indicator for the SDS-PAGE run with distilled water to 200 mL), first with buffer plus DTT (10 mL buffer + 100 mg DTT) for 15 min, then with buffer plus iodacetamide (IAA) (10 mL buffer + 250 mg IAA) for 15 more minutes. Thereafter, the strips were washed with 1× SDS buffer. After these preparation steps, the strips were loaded on gels and covered with agarose solution (125 mg agarose, 50 µL 1% bromphenol solution, 125 mL 1× SDS buffer). The electrophoresis chambers (Protean^®^ SDS-PAGE cell, BioRad, Hercules, CA, USA) were both filled with 1× SDS-buffer. SDS-PAGE was then conducted at 32 mA per gel for 45 min, followed by 48 mA per gel for approximately five hours (until the bromphenol reached 1 cm distance from the lower margin of the gel).

Every gel was loaded with one aliquot of the standardized pool sample and CSF-samples of two single patients—separate for CIS- and control patients on different gels.

These gels were scanned with Typhoon^®^ 9410 scanner (Amersham, Little Chalfont, UK) which transferred data to the DeCyder^®^ version 6.5 2D software for statistical analyses.

### 2.3. Statistics

Gels were analyzed using DeCyder^®^ 6.5 software (GE Healthcare, Little Chalfont, UK). We performed in-gel analyses of spot patterns (each spot numbered by the software due to its position on the gel) comparing the number and protein level of spots between the samples loaded on the same gel. Furthermore, batch-analyses were performed in order to compare match of spots between samples on different gels. All detected spots with peak height <30 units were excluded because such low signals are unlikely to represent true protein spots. To classify spots as different between two samples, we chose a threshold of 2.0-fold standard deviation (similar to Lehmensiek et al. [13]). The numbers and percentages acquired during spot pattern comparison were presented with descriptive statistics in the results sections. For analysis of demographic variables we used D’Agostino-Pearson normality test and Mann-Whitney-test.

## 3. Results

The control group included seven patients with normal pressure hydrocephalus, one migraine, one stroke and one lumbago; in this group there were 7 female (70%) and 3 male (30%) patients, whereas the CIS group (clinically isolated syndrome, i.e., first relapse of relapsing-remitting MS) comprised from 13 female (65%) and 7 (35%) male patients. Median age of patients at time of sampling was 30 (24–48) years in the CIS-group and 75 (48–90) years in the control-group (*p* < 0.001). CSF characteristics of both groups are displayed in Table 1.

We had to exclude six samples (all of the CIS-group) were the DeCyder^®^ software was not able to identify all spots due to insufficient protein separation resulting in 14 CIS samples and 10 control samples for further analyses. Altogether, 273 ± 12 different spots were detected per gel, 127 ± 44 of those were excluded because they did not fulfill typical properties of a protein spot resulting in a mean of 146 ± 40 spots per gel remaining for further analyses.

### 3.1. Intra-Gel Analysis

We compared samples within the single gels which all were labeled with one pool (compound of all CIS and all control samples, respectively) and two single patients’ samples. These results are listed in Table 2. The median match (with ranges) of protein spots of two samples within one gel, that is, the spots not exceeding a difference of two standard deviations of protein level, was 89.7% (68.4–93.4%) in the CIS-group and 94.9% (93.2–98.2%) in the control group. By including one sample (no. 13) where protein separation was not optimal, but still detectable by the software, we showed susceptibility of the method on variations and small inaccuracies of test performance. In this case, a slightly lower protein separation resulted in about 32% of spots not matching in their protein level within one single gel. When excluding this sample form analysis, the median percentage for matching spots within the CIS-group was 89.8% (82.8–93.4%) with no significant difference compared to the control group. Additionally, we compared all single samples with the pool sample within every single gel. The median percentage of matching protein spots was 95.4% (81.1–99.4%—excluding the one sample with low protein separation) for CIS and 94.9% (84.3–98.0%) for controls (*p* = 0.84). In order to investigate the influence of Cy-labeling, two samples (CIS no. 9 and 10) were cross-labeled with the opposite Cy-dye and compared with the same pool sample, resulting in comparable results for both gels (see table). Also, matching of samples labeled with Cy3 (all uneven sample numbers) and Cy5 (even sample numbers) with the pool sample (always labeled with Cy2) did not show any difference.

### 3.2. Inter-Gel Analysis

As a second step, we performed batch analyses, where protein spots in different gels where compared. Dependent on which particular gels where matched, we found between 4 and 13 spots per analysis with different protein level. Table 3 shows exemplary match pairs within the control group (comparable results for CIS-patients) with absolute number of spots with different protein level between Batch A and B. Additionally, we matched all samples of the CIS and control group labeled with Cy3 with all samples labeled with Cy5. Here, number of different spots between two batches varied noticeably between Cy3- and Cy5-labelled samples. Moreover, the table shows batch analyses stratified by sex and age, whereby we were not able to detect any differences regarding sex, whereas, some influence of age on number of different spots per match pair cannot be definitely excluded. Eventually, we matched all CIS patients against all control patients and found 28 spots of different protein level, which may be of interest for finding differences in protein synthesis in CSF between MS and non-MS patients.

For further analyses all detected spots were numbered. Table 4 shows examples of batches with the numbers of those spots which were identified as different between two particular batches, including the above described 28 spots which were identified as to be different in comparison of all CIS versus all control patients. In various comparative analyses we showed that we were able to find an amount of different spots in several combination of gels, however, the number of spots identified as different varied dependent on which combination of gels were matched. For example, we matched the CIS-samples no. 3 and 4 with all gels of control patients. The total amount of spots of different protein level between these CIS samples and the particular control samples seems constant (10 to 13), however, for every match of gels different single spots were calculated to have a different abundance as shown by listed spot numbers. The table includes further examples for other batch pairs which showed similar results. Therefore, if one intends to pick proteins for further analyses, every comparison of different gels results in different protein spots to seem to be of interest for spot picking. In the above-described example only spot no. 14 was consistent through all possible comparisons with different control patients, all other spots could not be identified as different in all possible gel combinations. Similar results were obtained for other batch combinations.

## 4. Discussion

In the present study, we aimed to analyze limitations of 2D-DIGE in proteomic studies of the CSF in order to explore possible reasons why results of previous studies in MS patients have been so controversial [5,6,7,8,9,10,11,12,13]. It seems obvious using 2D-DIGE for identification of proteins in the CSF of MS patients. It is a high-resolution method allowing protein separation by molecular weight and charge with consequent spot picking from the gels. By that, one can visualize differently expressed proteins in different patients or cohorts and identify these e.g., with mass spectrometry. In contrast to conventional 2D gel electrophoresis with silver staining, where methodological variation by running samples on different gels can be a significant limitation, 2D-DIGE allows the comparison of samples processed within the same gel under the same conditions [13]. However, one disadvantage of 2D-DIGE is still the lack of a standardized protocol [6]. There are many methodological steps where different reagents, buffers and laboratory devices are used, influencing not only the quality of the final protein separation, but also the protein spots to be detected, that may result in discrepant proteins identified as differentially expressed between cohorts. One described example for this methodological issue is Cystatin C, which was first identified as a potential biomarkers specific for MS, then considered as a storage artifact by other authors [19,20,21].

During validation of the method in our laboratory we observed a high susceptibility of 2D-DIGE for methodological variations leading to very different patterns even if using the same CSF samples. Although we used the above described protocol for all samples and processed them in one run, at the end we had to exclude four samples due to low protein separation which did not allow further analyses with DeCyder software. One sample (CIS no. 13) could be analysed by the software, but yielded 32% of spots with different protein separation compared to another CIS sample run on the same gel, again because of low protein separation. For our analyses, we chose very restrictive criteria for protein spot identification in order not to include detected spots which potentially do not represent a specific protein, yielding a comparatively low number of spots. Considering the complicated and error-susceptible technique we estimate major limitations of the so far established 2D-DIGE protocols regarding their utility as an explorative tool for identifying possible biomarkers for MS. A standardized protocol or even automated performance of the assay could limit this problem.

Another problem influencing results of 2D-DIGE crucially is CSF collection itself. Blood contamination during lumbar puncture would lead to a significant change in protein concentration in CSF, since protein concentration in the blood is hundred- to thousand-fold higher compared with CSF, so that even slight blood contamination would change results of a study performed in the CSF [12]. Therefore, we only used samples without blood contamination for both groups. Another pre-analytical confounder that has to be considered is, whether CSF collection was performed before or during an intravenous cortisone therapy [9] as this could influence presence of particular proteins as well.

Another issue of previous studies was the use of pooled samples (such as in Lehmensiek et al. and Li et al. [11,13]) for detection of differently expressed proteins in MS and control samples. The arguments for using pooled instead of single samples are the low protein concentration in CSF, allowing higher protein quantity when using higher amounts of CSF which are often not available from one single patient, and, furthermore, authors suggest reducing individual variability of the proteome when using pooled samples. Therewith, a better representative analysis for a larger patients group should be obtained. When analyzing percentage of different spots among single patients and between single and pooled samples, we showed a variation of about 10% of protein spots identified as different in patients run on the same gel. This means that 10% of spots appear as differently expressed proteins due to inter-individual variability of protein expression only and not due to disease-specific changes of protein expression. Therefore, the results of 2D-DIGE studies may vary dependent on which CSF samples are chosen for the study and used for the pool. Although changes of CSF proteome in MS have to be assumed [6,13], it seems unlikely that a heterogeneous disease like MS should lead to the same changes of protein expression in all patients. This finding underlines the problem of using 2D-DIGE in pooled patients.

Again focusing on methodological variations, we showed that the number of spots identified as different varies when comparing sample batches on different gels. In different comparative analyses we found 4 to 30 differently expressed protein spots which could be picked for identification. Also, the number of detected spots was different in samples labeled with different Cy-dyes, so that the labeling process could influence the results as well. As a further step, we numbered the spots in all gels and analyzed, whether the numbers of spots with different protein levels vary between different batches. Table 4 gives examples of batches with spot numbers which were identified as possible candidate markers due to a difference of protein quantity of more than two standard deviations. Although detecting up to 30 differently expressed spots, there was only one spot for samples CIS3+4 which was identified as different in all possible comparisons with control samples. Using other CIS samples, other spots seemed to represent possible specific proteins with different expression in CIS patients. This finding finally explains the variability of candidate proteins in different studies. The variations due to individual protein expression and due to methodological variations seem that high, that consistent results throughout a large number of samples and gels with different samples compared in the consecutive statistical analysis could not be suspected.

While 2D-DIGE alone with its current possibilities and flaws seems not to be an ideal tool for diagnosis of MS, several serum and CSF proteins have been investigated using other assay methods in order to find possible biomarkers in MS. For example, a current study found elevated levels of caspase-1, apoptosis-associated speck-like protein containing a caspase recruitment domain and interleukin 18 to be associated with MS [22]. Many studies showed promising results regarding CSF biomarkers. One of the currently most investigated markers is neurofilaments. Similarly, chemokines such as CXCL-13 (C-X-C motif chemokine 13) as well as Fetuin A and glial fibrillary acidic protein seem elevated in CSF of MS patient as well, suggesting these molecules as potential biomarkers for disease activity and/or progression [23]. Another study showed that the CSF protein 14-3-3 could indicate more severe disease courses in demyelinating diseases [24]. Such biomarkers, if confirmed in larger study designs, could improve the diagnostic process in MS and, moreover, could be of interest in future approaches of 2D-DIGE techniques as well in order to combine these methods for improving sensitivity and specificity.

Another issue to discuss is the dependency of CSF proteomics from neuronal cell death. Present literature indicates that acute phase proteins, e.g., plasma Sirtuin 1, could contribute information in order to quantify neuronal damage in neurodegenerative diseases. Markers for neuronal damage could help interpreting protein patterns in 2D gels by combining such analyses [25].

Taken together, we assume that special biomarker analyses in serum and/or CSF in combination with 2D-DIGE patterns could help to improve the utility of the method in further scientific approaches by reducing methodological variation between different studies, however, one has to take into account that the vast heterogeneity of the disease may cause a significant variation of all potential markers making diagnosis by one single method difficult or even impossible. Therefore, difficulty in CSF analysis is not a specific problem of 2D-DIGE, but of other techniques such as immunoblot etc. as well.

There are limitations of our study. Similar to other studies there may have been methodological problems as described above. We tried to reduce influence of low protein separation on statistical analyses by considering well-separated protein patterns only. In order to increase protein concentration we did not deplete albumin and immunoglobulins in the CSF since this would lead to a markedly lower concentration of other proteins as well. The commercially available kits for albumin and immunoglobulin depletion have been developed for serum and plasma, whereas they are not compatible with the low protein concentration in the CSF [11,26].

Moreover, Fodor et al. [27] discussed issues of statistical analyses with DeCyder software which have to be considered.

Another limitation to discuss is the significantly different age between MS patients and control subjects. This problem arises due to the fact that diagnosis of multiple sclerosis is the most common indication for lumbar puncture in young people, whereas, most of neurologic symptoms which lead to lumbar puncture in clinical routine develop in much older patients. Therefore, it is difficult to get CSF from young symptomatic controls as defined by a consensus paper [18]. We tried to show batch analyses stratified by age and sex where possible, however, a major influence especially by age cannot be definitely ruled out. Indeed, we emphasize that our study was not designed for age-dependent analyses.

Our study was not designed for identification of single proteins because we wanted to explore the diagnostic utility of 2D-DIGE in multiple sclerosis and because of controversial results of spot picking in previous studies. We were particularly interested in showing inter-individual variations and susceptibility of the method itself for potential errors.

With the here shown results regarding variation and precision of the method identification of specific CSF proteins being predictive for MS, i.e., which can be identified as a surrogate marker in all possible gel combinations, seems difficult. Therefore, a standardized 2D-DIGE protocol is highly recommended in order to reduce methodological variation among different studies. In addition, variable protein expression in single subjects has to be taken into account since this might confound results when pooling CSF of different patients.

## Figures and Tables

**Table 1:** Cerebrospinal fluid (CSF) characteristics of control- and clinically isolated syndrome (CIS)-patients.

CSF Parameter	Value	*p*-Value
**CSF/serum glucose ratio**		
controls	0.64 ± 0.13	
CIS	0.65 ± 0.07	0.584
**total CSF protein (mg/dL)**		
controls	38.5 (27–58)	
CIS	35 (21–75)	0.251
**erythrocyte count (cells/µL)**		
controls	0 (0–322)	
CIS	1 (0–49)	0.378
**white blood cell count (cells/µL)**		
controls	1 ± 1	
CIS	12 ± 9	0.005

All CIS-patients had positive oligoclonal bands, whereas all control patients had none.

**Table 2:** Percentage of spots with similar protein level in samples within single gels.

(IN-GEL ANALYSIS)	Spots with Similar Protein Concentration	Spots with Different Protein Concentration >2 SD
A		
CIS-patients		
1 vs. 2	89.7%	10.3%
3 vs. 4	89.9%	10.1%
5 vs. 6	90.5%	9.5%
7 vs. 8	82.8%	17.2%
9 vs. 10	89.0%	11.0%
11 vs. 12	93.4%	6.6%
13 * vs. 14	68.4%	31.6%
B		
control patients		
1 vs. 2	94.9%	5.1%
3 vs. 4	93.2%	6.8%
5 vs. 6	98.2%	1.8%
7 vs. 8	96.2%	3.8%
9 vs. 10	94.8 %	5.2%
C		
POOL CIS vs. single CIS-patients		
Pool vs. 1	84.8%	15.2%
Pool vs. 2	95.4%	4.6%
Pool vs. 3	81.1%	18.9%
Pool vs. 4	92.3%	7.7%
Pool vs. 5–8	(analysis not performed due to software incompatibility of pool image)	
Pool vs. 9	93.3%	6.7%
Pool vs. 10	96.1%	3.9%
Pool vs. 9 (2nd gel) **	94.4%	5.6%
Pool vs. 10 (2nd gel) **	95.5%	4.5%
Pool vs. 11	97.9%	2.1%
Pool vs. 12	99.4%	0.6%
Pool vs. 13 *	68.0%	32.0%
Pool vs. 14	95.8%	4.2%
D		
POOL control vs. single control patients		
Pool vs. 1	85.8%	14.2%
Pool vs. 2	92.9%	7.1%
Pool vs. 3	84.3%	15.7%
Pool vs. 4	96.5%	3.5%
Pool vs. 5	91.4%	8.6%
Pool vs. 6	96.5%	3.5%
Pool vs. 7	98.0%	2.0%
Pool vs. 8	97.2%	2.8%
Pool vs. 9	96.0%	4.0%
Pool vs. 10	93.7%	6.3%

The percentages of spots that show similar protein level between (A) the CSF of two single CIS patients, (B) the CSF of two single control subjects, (C) the single and pooled CSF of CIS patients and (D) the single and pooled CSF of control subjects are shown. SD, standard deviation. * low quality of protein separation, ** two CIS CSF were run twice in two different gels with opposite Cy-labeling.

**Table 3:** Number of spots with different protein level in various matched sample batches between different gels.

Batch A	Batch B	Number of Different Spots
controls 1+2	controls 3+4	10
controls 1+2	controls 5+6	8
controls 3+4	controls 5+6	4
controls 3+5	controls 4+6	6
controls 1+3	controls 2+4	6
controls 1+3+5+7+9	controls 2+4+6+8+10	6
controls 1+3+5	controls 2+4+6	7
controls 1+3+5+8+10	controls 2+4+6+7+9	5
controls 2+3+6+7+10	controls 1+4+5+8+9	10
controls 5+6	controls 7+8	13
controls 5+7	controls 6+8	5
controls 3+4+5+6	controls 7+8+9+10	11
CIS 1+3+9+11+13 *	controls 1+3+5+7+9 *	30
CIS 2+4+10+12+14 **	controls 2+4+6+8+10 **	8
all CIS	controls 6+8+9 (male)	24
all CIS	controls 1+2+3+4+5+7+10 (female)	28
CIS 1+2+3 (male)	controls 6+8+9 (male)	18
CIS 4+10+13 (female)	controls 6+8+9 (male)	11
all CIS	controls 1+8 ***	21
all CIS	controls 2+3+4+5+6+7+9+10	27
CIS 3+4	Controls 1+8 ***	8
CIS 3+4	Controls 6+10	15
all CIS	all controls	28

* all samples labeled with Cy3. ** all samples labeled with Cy5. *** age-matched controls (age 48 and 49).

**Table 4:** Proteins identified with different level by spot number, shown in exemplary matched batch pairs.

CIS 3+4	Number of Different Spots	Spots with Different Protein Level >2 SD
controls		
1+2	12	14, 15, 23, 32, 36, 51, 55, 73, 125, 161, 168, 179
3+4	13	14, 15, 22, 25, 32, 36, 51, 74, 124, 125, 172, 177, 179
5+6	12	14, 15, 32, 36, 73, 85, 110, 116, 124, 125, 128, 179
7+8	10	14, 15, 36, 138, 155, 161, 168, 169, 172, 174
9+10	11	14, 32, 43, 51, 68, 79, 85, 126, 171, 172, 179
1 to 10	11	14, 15, 32, 36, 51, 85, 128, 161, 172, 177, 170
CIS 1+2		
controls		
1+2	9	2, 14, 40, 50, 55, 61, 79, 125, 145
3+4	14	18, 25, 32, 40, 57, 61, 69, 74, 81, 124, 125, 144, 147, 177
5+6	15	40, 44, 85, 102, 110, 116, 121, 124, 125, 127, 128, 132, 144, 186, 188
7+8	12	11, 16, 19, 38, 40, 44, 57, 86, 95, 127, 138, 144
9+10	12	26, 40, 43, 68, 79, 98, 124, 126, 132, 172, 176, 189
1 to 10	14	2, 11, 16, 40, 44, 61, 124, 125, 126, 127, 128, 132, 147, 177
CIS 11+12		
controls		
1+2	6	14, 35, 55, 92, 105, 121
3+4	5	7, 74, 76, 92, 99
5+6	3	85, 116, 144
7+8	6	19, 92, 95, 104, 121, 142
9+10	6	43, 59, 68, 98, 106, 181
1 to 10	2	7, 92
all CIS		
all controls	28	1, 11, 12, 14, 16, 25, 32, 35, 36, 42, 44, 48, 61, 109, 133, 137, 140, 146,
		149, 152, 154, 162, 164, 172, 177, 179, 184, 188

CIS-sample pairs in bold were matched with control-sample pairs as indicated in the same column below. For each match, the count of different spots and specific numbers of spots identified as different, are shown.
